# Alopecia areata incognita: clinical characteristics and use of the Sinclair shedding scale

**DOI:** 10.1097/JW9.0000000000000067

**Published:** 2022-12-23

**Authors:** Maya S. Collins, Shaheir Ali, Kristen Kelley, Maryanne Makredes Senna

**Affiliations:** a Department of Dermatology, Lahey Hospital & Medical Center, Burlington, Massachusetts; b Harvard Medical School, Boston, Massachusetts

**Keywords:** alopecia areata incognita, alopecia, hair, treatment

**What is known about this subject with respect to women and their families?**

- Alopecia areata incognita (AAI) is a form of Alopecia Areata that presents as persistent, diffuse hair shedding without glabrous patches of hair loss.
- Patients maintain their normal hair density unless AAI has persisted for several years.
- AAI is a very challenging diagnosis to elucidate due to its resemblance to telogen effluvium. It is frequently very distressing for patients.
- Several AAI publications emphasize diagnosis based on follicular growth phases and trichoscopy.

**What is new in this article with respect to women and their families?**

- Herein, we describe a clinical phenotype for AAI.
- We demonstrate the utility of the Sinclair Shedding Scale for diagnosing AAI and monitoring the clinical course overtime.
- We also briefly describe our approach to treatment using intralesional corticosteroid injections and our patients’ subsequent response.

## Dear Editors,

Introduced in 1987, alopecia areata incognita (AAI), is incredibly difficult to diagnose. It presents as persistent, diffuse hair shedding while patients maintain normal hair density.^1,2^ We report 4 cases of AAI diagnosed in our specialty alopecia clinic, highlighting aspects of the condition that impart high levels of diagnostic suspicion.

A chart review conducted between December 2017 and March 2022 yielded 4 patients with AAI. All patients were Caucasian females, average age of 45.3 years (SD = 10.9), with several years of excess scalp hair shedding. They denied telogen effluvium (TE) triggers or patchy hair loss. Initial differential diagnoses included female pattern hair loss (FPHL) and chronic telogen effluvium. Despite diligent treatment with >6 months oral spironolactone and/or oral low dose minoxidil, patients reported no improvement in shedding.

**Fig. 1.**
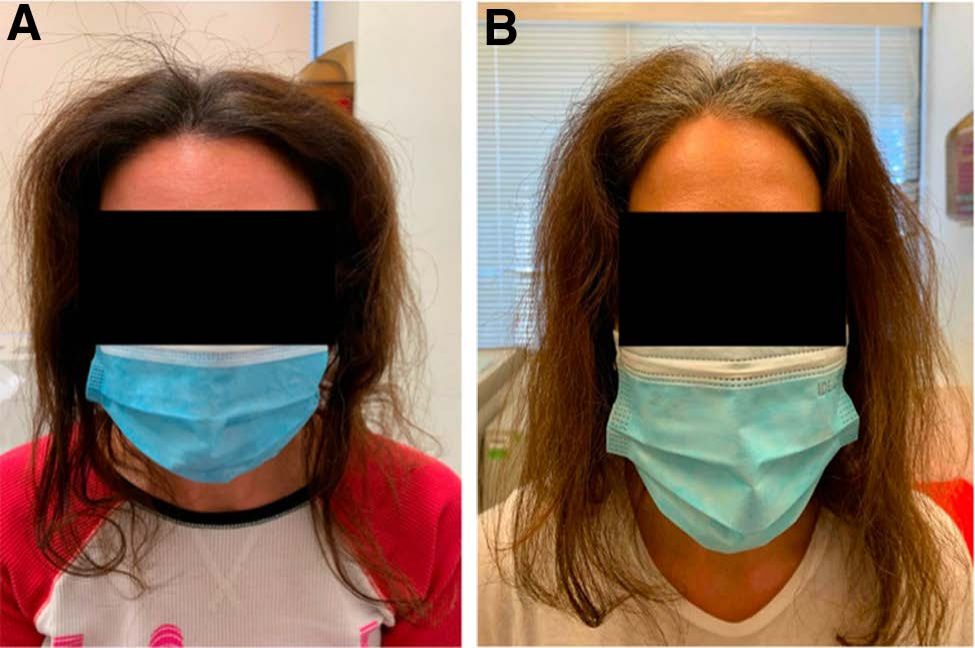
(A) Image showing patient with AAI before starting ICS injections. (B) Image showing patient with AAI after ICS injections demonstrating increased distal hair density. AAI, alopecia areata incognita; ICS, intralesional corticosteroid.

All patients were noted to have a distinctive global appearance: normal density of 5–6-cm length terminal hairs on the top of the scalp and dramatically decreased density of longer terminal hairs distally (Fig. 1). Trichoscopy was unrevealing. AAI patients may develop circular patches of classic alopecia areata, but this was not observed in our initial evaluations.^2,3^

Scalp biopsies in all 4 patients varied with findings and the respective dermatopathologist favored diagnoses are listed in Table 1. Biopsy findings in AAI are subtle and vary depending on the disease stage.^4,5^ Histopathology in our patients was insufficient for a definitive diagnosis of alopecia areata incognita.

**Table 1:** Histopathologic findings in biopsies of patients with AAI

Histopathologic finding	Number of patients with finding	Dermatopathologist favored histopathologic diagnoses
Increased number of early telogen follicles	2	AAI
Decreased or atrophic sebaceous glands	2	CAA or FPHL
Mild peribulbar inflammation	1	AAI or TE
Fibrous streamers	2	CAA

AAI, alopecia areata incognita; CAA, classic alopecia areata; FPHL, female pattern hair loss; TE, telogen effluvium.

Patients with AAI may collect 350–1000 hairs per day using the modified wash test.^6^ As the modified wash test is notably time consuming and difficult, we utilized the Sinclair shedding scale (SSS) to assess hair shedding.^7^ All patients reported >Grade 6 daily shedding on the SSS, representing about 750 hairs per day.^7^

Given our suspicion for AAI, we added monthly intralesional corticosteroid scalp injections at 5 mg/mL doses across the scalp. Patients did not use adjunct topical corticosteroids. After 2 treatment sessions, all patients reported SSS score improvement to the normal range and increased distal hair density (Fig. 1). One patient developed a few patches of subcentimeter classic alopecia areata that subsequently improved.

Several AAI publications emphasize diagnosis based on growth phases and trichoscopy.^2,3^ We highlight an AAI clinical phenotype and demonstrate utility of the SSS when AAI is suspected. Originally developed to aid diagnosis of FPHL, the SSS is useful in assessing the response to treatment in women with varying hair lengths. Given that our patients had straight hair without improved SSS scores despite adequate treatment of FPHL, the SSS provided critical clinical estimates of shedding without extensive effort from patients or providers. The SSS was also useful in tracking normalization of hair shedding after monthly intralesional corticosteroid in our patients, and this correlated with increased hair length for our patients.

AAI is a challenging diagnosis, rarely supported by scalp biopsy findings. We hope this report highlights additional clinical indications that may aid in making the diagnosis.

## Conflicts of interest

None.

## Funding

MMS was supported by clinical and research salary for this study.

## Study approval

N/A.

## Author contributions

MSC participated in research design, in the writing of the paper, in the performance of the research, and in data analysis. SA participated in the writing of the paper. KK participated in the writing of the paper. MMS participated in the research design, in the writing of the paper, in the performance of the research, and in data analysis.

## Consent

Informed, written consent was received from all patients and confirmed to the journal pre-publication, stating that the patients gave consent for their photos and case history to be published.
